# Subgroup-Enriched Pathways and Kinase Signatures in Medulloblastoma Patient-Derived Xenografts

**DOI:** 10.1021/acs.jproteome.2c00203

**Published:** 2022-08-17

**Authors:** Kristin L. Leskoske, Krystine Garcia-Mansfield, Ritin Sharma, Aparna Krishnan, Jessica M. Rusert, Jill P. Mesirov, Robert J. Wechsler-Reya, Patrick Pirrotte

**Affiliations:** † Cancer and Cell Biology Division, Translational Genomics Research Institute, Phoenix, Arizona 85004, United States; ‡ Integrated Mass Spectrometry Shared Resource, City of Hope Comprehensive Cancer Center, Duarte, California 91010, United States; § Tumor Initiation and Maintenance Program, NCI-Designated Cancer Center, Sanford Burnham Prebys Medical Discovery Institute, La Jolla, California 92037, United States; ∥ Department of Medicine, University of California San Diego, La Jolla, California 92093, United States; ⊥ Moores Cancer Center, University of California San Diego, La Jolla, California 92093, United States

**Keywords:** medulloblastoma, pediatric, brain tumor, proteomics, kinase activity, patient-derived xenograft (PDX), actinomycin D, MYC

## Abstract

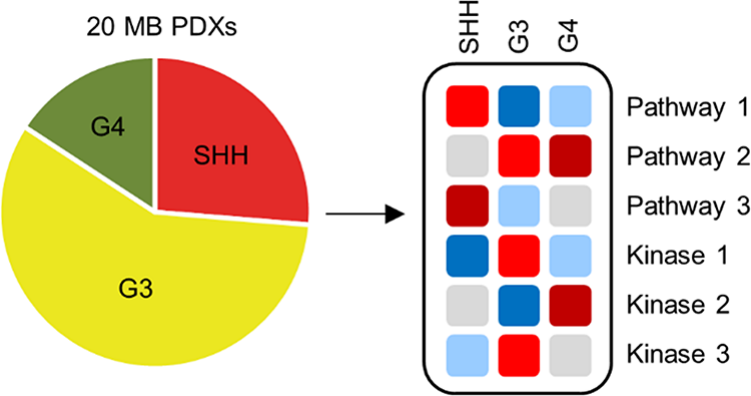

Medulloblastoma (MB) is the most common malignant pediatric brain tumor. MB is classified into four primary molecular subgroups: wingless (WNT), sonic hedgehog (SHH), Group 3 (G3), and Group 4 (G4), and further genomic and proteomic subtypes have been reported. Subgroup heterogeneity and few actionable mutations have hindered the development of targeted therapies, especially for G3 MB, which has a particularly poor prognosis. To identify novel therapeutic targets for MB, we performed mass spectrometry-based deep expression proteomics and phosphoproteomics in 20 orthotopic patient-derived xenograft (PDX) models of MB comprising SHH, G3, and G4 subgroups. We found that the proteomic profiles of MB PDX tumors are closely aligned with those of primary human MB tumors illustrating the utility of PDX models. SHH PDXs were enriched for NFκB and p38 MAPK signaling, while G3 PDXs were characterized by MYC activity. Additionally, we found a significant association between actinomycin D sensitivity and increased abundance of MYC and MYC target genes. Our results highlight several candidate pathways that may serve as targets for new MB therapies. Mass spectrometry data are available via ProteomeXchange with identifier PXD035070.

## Introduction

Medulloblastoma (MB) comprises a heterogeneous group of malignant brain tumors that most commonly occur in children. Genomic and transcriptomic analyses have identified four major subgroups of MB that differ in molecular features and patient outcomes: wingless (WNT), sonic hedgehog (SHH), Group 3 (G3), and Group 4 (G4).^1^ WNT MB has the best prognosis, with 5 year survival rates >90%.^2^ Most WNT tumors contain activating mutations in CTNNB1, which codes for β-catenin.^3^ Likewise, many SHH tumors carry mutations in genes encoding key members of the SHH signaling pathway.^3^ SHH MB has an intermediate prognosis, and genomic events such as loss of function mutations in TP53 are associated with poor outcome.^4^ While WNT and SHH MB are characterized by aberrant activation of their namesake developmental pathways, less is known about the signaling pathways driving G3 and G4 MB. G3 tumors are frequently metastatic and have a poor prognosis with 5 year survival rates of approximately 50%.^5^ The most common molecular alteration in G3 MB is amplification of MYC, which occurs in approximately 20% of G3 tumors^3^ and is associated with poor clinical outcomes.^6^ G4 MB is the most common type of MB and has an intermediate prognosis.^5^ While recurrent mutations have been identified in G4 MB, they occur at relatively low frequencies.^3^ Recently, ERBB4-SRC signaling was identified as a potential driver of G4 MB.^7^

Proteomic and phosphoproteomic analyses have shown that WNT, SHH, G3, and G4 MB display distinct proteomic features.^7,8^ Two proteomic subtypes of SHH and G3 MB, termed SHHa and SHHb, and G3a and G3b, have been reported.^8^ Compared to SHHb MB, SHHa MB is enriched for proteins involved in RNA processing, MYC pathway, and chromatin modification. In contrast, SHHb tumors are enriched for proteins involved in neuronal and glutamatergic synapse signaling. G3a MB is characterized by MYC activation via amplification or post-translational modification. The primary pathways driving G3b MB are unknown.

Current treatment strategies for MB include surgical resection, craniospinal radiation, and multi-agent chemotherapy. However, patient outcomes vary depending on clinical and molecular features. Additionally, many MB survivors experience lifelong side effects from their treatment, emphasizing the need for more effective and less toxic therapies.^9^ Pre-clinical drug development requires well-characterized disease models that closely resemble primary human tumors. We recently performed high-throughput drug screening on 20 orthotopic patient-derived xenograft (PDX) models of MB comprising SHH, G3, and G4 subgroups and identified subgroup-specific drug sensitivities including actinomycin D as a potential targeted therapy for G3 MB.^10^ In this study, we sought to characterize the proteomic features and phospho-signaling pathways enriched in these 20 PDX models as well as identify proteomic signatures associated with drug sensitivity.

## Experimental Procedures

### Experimental Design and Statistical Rationale

The proteomes and phosphoproteomes of 20 orthotopic patient-derived xenograft (PDX) models of medulloblastoma (MB) were analyzed by LC–MS/MS. Our cohort (Table S1) contains multiple PDXs representing the three most prevalent subgroups of medulloblastoma: SHH (*n* = 6), G3 (*n* = 10), and G4 (*n* = 4). MYC-amplified G3 lines are overrepresented in our cohort because this subtype is known to have poor clinical outcomes. Due to limited sample material, technical replicates were not performed. The 20 PDX samples were randomly assigned to one of two TMT 11-plexes (Table S2) each comprising 10 PDX cell lines and an internal reference consisting of equal amounts of peptide from all 20 PDX lines. Med-1911FH was determined to be an outlier and excluded from statistical calculations. Due to small and uneven sample group sizes, differences in protein abundance, phosphosite abundance, and kinase activity between subgroups were calculated using one-way ANOVA (*aov* function in the R stats package coupled with the *ANOVA* function within car 3.0–8)^11^ with Tukey post-hoc and Benjamini–Hochberg correction for multiple hypothesis testing (*TukeyHSD* function from the stats package). An adjusted *p*-value *q* < 0.05 was considered significant unless stated otherwise. The significance of differences in actinomycin D IC50 values between cell lines was determined by Student’s *t*-test using the *t.test* function in R.

### Animals

NOD-SCID IL2R-gamma null (NSG) mice used for intracranial tumor transplantation were purchased from Jackson Labs (Bar Harbor, ME). Mice were maintained in the animal facilities at the Sanford Consortium for Regenerative Medicine. All experiments were performed in accordance with national guidelines and regulations and with the approval of the animal care and use committees at the Sanford Burnham-Prebys Medical Discovery Institute and University of California San Diego (UCSD).

### Establishment and Maintenance of PDXs

PDX lines were generated by implanting 0.5–1 × 10^6^ dissociated patient cells directly into the cerebellum of NSG mice and propagated from mouse to mouse without in vitro passaging. The identity and subgroup of each line were validated by DNA methylation analysis. For proteomic studies, cells were isolated from tumor-bearing mice, washed at least twice with PBS, pelleted, and snap frozen in liquid nitrogen.

### Protein Digestion

PDX cells were lysed with urea lysis buffer (8 M urea, 75 mM NaCl, 50 mM Tris pH 8, 1 mM EDTA, and 1× HALT Protease and Phosphatase Inhibitor Cocktail (Thermo Fisher Scientific)) and sonicated. Lysates were clarified by centrifugation at 20,000*g* for 10 min. Protein concentration was quantitated using a Pierce BCA Protein Assay Kit (Thermo Fisher Scientific). Equal amounts of protein (296 μg) from each sample were reduced with 5 mM dithiothreitol for 45 min and alkylated with 10 mM iodoacetamide for 45 min in the dark prior to digestion with lysyl-endopeptidase (1:100 enzyme:protein) (FUJIFILM Wako Pure Chemical Corporation) for 4 h followed by overnight digestion with Trypsin Gold (1:50 enzyme:protein) (Promega). Digests were acidified with formic acid and centrifuged at 2000*g* for 5 min to remove the precipitate. Peptides were desalted with 100 mg C18 cartridges (Waters).

### Isobaric Labeling

150 μg of peptide from each sample was labeled with an 11-plex tandem mass tag (Thermo Fisher Scientific) according to the manufacturer’s protocol. 1 μg of peptide from each TMT labeling was analyzed by mass spectrometry to ensure >99% labeling efficiency. TMT labeling reactions were quenched with hydroxylamine, pooled, and desalted with 200 mg C18 cartridges (Waters).

### Phosphoenrichment

Phosphopeptides were enriched by sequential metal oxide affinity chromatography.^12^ Briefly, phosphopeptides were enriched using a High-Select TiO_2_ Phosphopeptide Enrichment Kit (Thermo Fisher Scientific). TiO_2_ flow-through and wash fractions were combined and further enriched using a High-Select Fe-NTA Phosphopeptide Enrichment Kit (Thermo Fisher Scientific). TiO_2_ and IMAC elutions were combined and fractionated into nine fractions using a Pierce High pH Reversed-Phase Peptide Fractionation Kit (Thermo Fisher Scientific) according to the manufacturer’s protocol with the addition of a ninth fraction at 100% acetonitrile. IMAC flow-through was used for global deep expression proteomics.

### Offline Fractionation

Pooled TMT labeled peptides were fractionated by high pH reverse phase chromatography performed using a Dionex U3000 HPLC system (Thermo Fisher Scientific). Peptides were loaded on a Waters XBridge C18 column (4.6 mm × 250 mm, 3.5 μm), and chromatographic separation was performed using a gradient of 96 min using a ternary solvent system A (water), B (acetonitrile), and C (50 mM ammonium hydroxide, pH 10) at a flow rate of 0.5 mL/min. Eluted peptides were collected every 60 s in a serpentine fashion on a 96 deep-well plate.^13^ The 96 fractions were condensed into final 24 fractions for global proteomics analysis by pooling every 24th fraction into a single fraction starting from the first fraction.^14^

### Data Acquisition and Protein Identification

Data acquisition was performed on an Orbitrap Fusion Lumos (ThermoFisher) mass spectrometer connected to a U3000 RSLCnano UHPLC system (ThermoFisher) utilizing water, 0.1% formic acid as Solvent A and acetonitrile, 0.1% formic acid as Solvent B. Dried peptides were reconstituted in 5 μL of LC–MS grade water with 2% acetonitrile and 0.1% formic acid and directly loaded on a 25 cm C18 column (2 μm particle size, 75 μm ID, EASY-Spray column, ThermoFisher) maintained at 45 °C. Peptides were eluted over 120 min by increasing the concentration of B as described: 2–19% in 80 min, 19–30% B in 20 min, 30–98% B in 5 min, maintaining 98% B for 2 min, 98% to 2% B in 1 min, and column equilibration for 11 min. Spectra were acquired in top-speed SPS-MS3 mode with MS1 in the Orbitrap (120 K resolution, 375–1500 *m*/*z* scan range, max injection time of 50 ms), MS2 of most abundant precursors in an ion trap (0.7 Da isolation window, CID fragmentation with 35% collision energy, activation time of 10 ms), and MS3 on MS2 fragments in the Orbitrap (2 Da isolation window, HCD fragmentation with 65% collision energy, 50 K resolution, max injection time of 50 ms, scan range of 100–500 *m*/*z*).^15^ A dynamic exclusion filter of 60 s was applied to prevent resampling of the same precursors.

A combined human and mouse protein database was generated by combining the UniProt^16^ human protein database downloaded on 03-29-2017 (42,150 entries) with the UniProt mouse protein database downloaded on 03-29-2017 (59,066 entries). Spectra were searched against the combined human and mouse protein database using Proteome Discoverer 2.2 software (Thermo Fisher Scientific) and the Mascot search engine (version 2.6) for global proteome fractions and the Byonic search engine (version 2.12) for phospho-enriched fractions. Up to two missed cleavages were allowed. Precursor mass tolerance was set to 10 ppm, and fragment mass tolerance was set to 0.6 Da. Carbamidomethylation of cysteine and TMT modification of lysine and any N-terminus were specified as fixed modifications. Methionine oxidation and N-terminal acetylation were set as dynamic modifications. Phosphopeptide searches also included serine, threonine or tyrosine phosphorylation as dynamic modifications with a maximum of four dynamic modifications. MS3 reporter ion peaks were integrated using the most confident centroid and an integration tolerance of 20 ppm. Peptide abundance was calculated using reporter ion peak intensity with a co-isolation threshold of 75 and average reporter S/N threshold of 10. Reporter ion isotopic impurities were corrected for using the correction factors provided in the TMT product data sheet. FDR was calculated by target decoy database search using the Percolator algorithm for global proteomics data and as part of the Byonic search algorithm for phosphoproteomics data. A target FDR of 0.01 was used to filter PSMs and peptides. Proteoclade^17^ was utilized to annotate peptides belonging to human, mouse, or shared; filter for *Homo sapiens* specific peptides; and recalculate protein abundances from the sum of only human-unique peptides. Mass spectrometry data have been deposited to the ProteomeXchange Consortium via the PRIDE^18^ partner repository with the dataset identifier PXD035070.

### Data Normalization

For deep expression proteomics, proteins with abundances missing in ≥50% of samples were removed. Protein abundances were normalized using sample-loading (SL) followed by internal reference standard (IRS) normalization coupled with trimmed mean of m samples (TMM).^19^

Human-unique peptides from the phospho-enriched dataset were filtered to retain those that included a phosphorylation site, were unambiguous and high confidence, and had a Byonic PSM confidence score > 200 in at least one TMT-plex. Phosphopeptides with ≥50% missing data were removed, and phosphopeptides abundances were then normalized by SL/IRS/TMM as described above.^19^ Phosphopeptides were not normalized to global protein abundance to allow for the inclusion of phosphoproteins observed in the phospho-enriched fractions and not in the global deep expression proteomics data. Phosphopeptides with multiple Master Proteins that were unable to be unambiguously assigned to a single protein were removed. Global modification positions were extracted for all phosphosites with an unambiguous phosphosite position using the peptide localized modification position and the amino-acid sequence “Position in Master Protein”. The phosphopeptides were then condensed to phosphosites by averaging the normalized abundances of all peptides containing a given phosphosite.^20^

### Sample Classification

Unsupervised clustering was performed using the pheatmap 1.0.12^21^ and stats packages in R version 4.2.0^22^ using Euclidean distancing and Ward’s clustering method. Principal component analysis was performed using the *prcomp* function from the stats package in R, with variable centering and scaling. Partial least squares discriminant analysis was performed using the *plsda* function from the MixOmics package v6.10.9 (www.mixomics.org),^23^ in R v4.2.0. The algorithm was set to regression analysis with four clusters, and all other parameters were left at the default value.

### NMF and Metagene Projection

To verify our classification model, we compared our proteomics data to previously published proteomics data on primary MB tumors (Archer dataset).^8^ WNT samples were removed and robust *z*-scores of significant proteins in the Archer dataset were input into NMFConsensus, a module in GenePattern,^24^ testing *k* = 2 through *k* = 10, to determine the optimal number of unsupervised clusters. All other values were left at the default values. Robust *z*-scores from all proteins in the Archer dataset and from our deep expression proteomics dataset were then input into the MetageneProjection module in GenePattern as the model and test sets, respectively. Based on the results from NMFConsensus, we set *k* = 5, and no model set refinement was performed. All other parameters were left at the default values.

### Gene Set Enrichment Analysis

Gene Set Enrichment Analysis^25^ was performed with ssGSEA2.0 in R (https://github.com/broadinstitute/ssGSEA2.0) using the Hallmark and C2 Canonical Pathways gene set collections from version 6.2 of the MSigDB.^26,27^ Proteins were ranked by taking the negative log10 of their *p*-value and multiplying by the sign of the log2 fold-change between the two groups being compared. Significantly enriched gene sets (*q* < 0.05) that were upregulated in a given subgroup for all comparisons were visualized using the Enrichment Map app in Cytoscape v3.8.2^28^ with an edge cutoff (similarity) of 0.375 to maximize the overlap between gene sets.

### Kinase-Substrate Prediction and Kinase Activity Scoring

The human PhosphoSitePlus (PSP) database^29^ downloaded 01-29-2018 was used to match observed phosphosites with their modifying kinase. The kinase-substrate prediction tool NetworKIN^30^ was used to predict new kinase-substrate interactions. NetworKIN confidence scores ranged from 0 to 228 for our dataset with higher scores indicating higher confidence. To determine an optimal score cutoff for NetworKIN predictions, NetworKIN was run against all phosphosites with a known modifying kinase in the human PSP database. PSP annotated kinase-substrate predictions above a given threshold were considered true positives, while PSP annotated predictions below the threshold were considered false negatives. Kinase-substrate predictions not in the PSP database were considered true negatives if the NetworKIN score was below the given threshold and false positives if the NetworKIN score was above the given threshold. Matthew’s correlation coefficient was then calculated to determine the optimal NetworKIN confidence score cutoff of 5.88. Only kinase-substrate predictions with a NetworKIN confidence score greater than 5.88 were considered for analysis. Kinases not identified in either the proteomic or phosphoproteomic datasets were removed.

Kinase activity was then scored for all 20 PDXs with IKAP^31^ using PSP annotated and NetworKIN predicted kinase-substrate interactions and the following parameters: 100 iterations; lower bound = 0; upper bound = 24. Kinase substrate interaction networks were generated in Cytoscape.^28^

### Drug Correlation Analysis

Drug sensitivity scores identified from our prior screen^10^ were correlated with protein abundance and phosphosite abundance using matched pairs and the Spearman correlation coefficient. Drug sensitivity scores represent the average percent viability of cells after drug treatment compared to control as measured in triplicate experiments. Med-1911FH was excluded from correlation calculations due to being a proteomic outlier. For drugs represented multiple times in the drug screen, correlations were calculated separately for each screen entry and the median correlation value was taken for each protein. Gene ontology (GO) enrichment was performed using ToppFun.^32^ Protein interaction network was generated using the String app in Cytoscape^33^ and the default confidence score cutoff of 0.4. Normalized enrichment scores were calculated for the identified ActD Sensitivity Signature using ssGSEA 2.0 in R.^25^

Quantitative proteomics data on Cancer Cell Line Encyclopedia (CCLE) cell lines were downloaded from elsewhere.^34^ Single sample GSEA (ssGSEA) was performed using ssGSEA 2.0 in R to calculate a normalized enrichment score (NES) for proteins found in the HALLMARK_MYC_Targets_v1 and HALLMARK_MYC_Targets_v2 gene sets (version 6.2)^27^ for each cell line. Actinomycin D (ActD) IC50 values on the CCLE cell lines were obtained from the Genomics of Drug Sensitivity in Cancer Project^35,36^ via the depmap portal. ActD was represented twice in the screen as Drug IDs 1811 and 1911. ActD IC50 values were compared between high (top 50 NES) and low (bottom 50 NES) signature expressing cell lines as well as between cell lines with high (top 50) and low (bottom 50) MYC protein abundance. Significance was determined by Student’s *t*-test using the *t.test* function in R.

## Results

### Proteomic Classification of 20 MB PDXs

**Figure 1:**
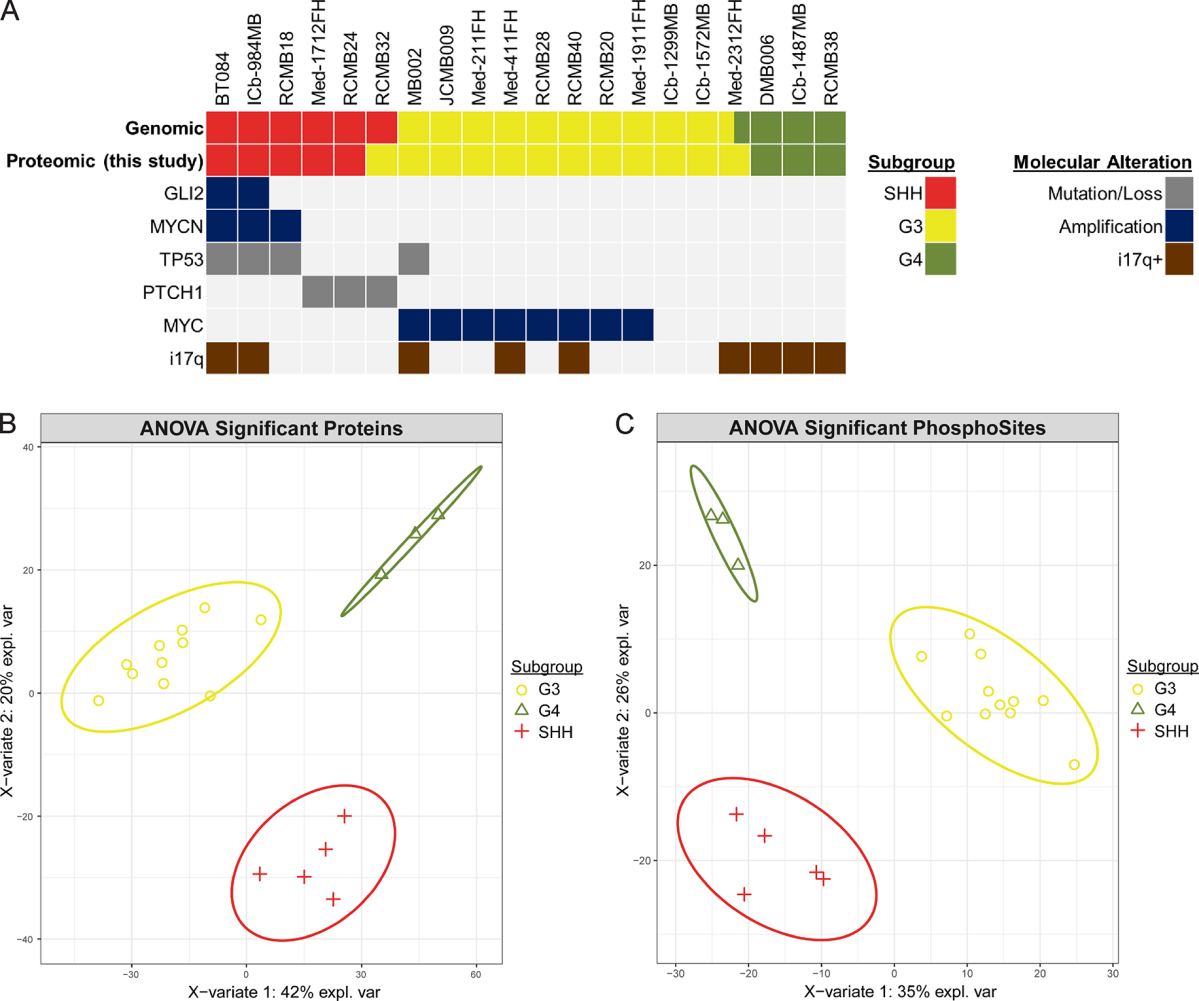
Sample overview and subgroup classification. (A) Common molecular alterations and subgroup classifications of 20 MB PDX models. (B) Partial least squares discriminant analysis on differentially abundant proteins (ANOVA *q* < 0.05) between subgroups. Ellipses indicate 95% confidence interval. (C) Partial least squares discriminant analysis on differentially abundant phosphosites (ANOVA *q* < 0.05) between subgroups. Ellipses indicate 95% confidence interval.

We acquired proteomic and phosphoproteomic data by liquid chromatography–tandem mass spectrometry on 20 PDX models of MB that were previously characterized and subtyped by DNA methylation and gene expression^10^ (Figure 1A). To minimize the influence of mouse stromal cell infiltration in our global deep expression proteomics and phosphoproteomics datasets, only peptides unique to human were used for analysis (Figure S1). This resulted in quantitative information on 6180 unique proteins and 4036 unique phosphosites (88.2% Ser, 11.8% Thr, 0.05% Tyr). Unsupervised clustering of proteomic and phosphoproteomic data showed that most PDXs clustered with their genomic subgroups (Figure S2). Med-2312FH, which has features of both G3 and G4 MB, consistently clustered with other G3 PDXs and was thus considered G3. G3 PDX Med-1911FH showed large variance by principal component analysis as well as more missing data (Figures S3 and S4). Thus, Med-1911FH was excluded from statistical calculations. RCMB32, which contains a PTCH1 mutation and was designated as SHH based on DNA methylation, consistently clustered with G3 and was included with G3 samples for statistical calculations. The remaining two p53 wildtype SHH PDXs (Med-1712FH and RCMB24) clustered separately from the p53 mutant SHH PDXs in the proteomics data but not in the phosphoproteomics data. Thus, p53 wildtype and p53 mutant SHH PDXs were not considered distinct proteomic subtypes. Partial least squares discriminant analysis showed that SHH, G3, and G4 PDXs contain distinct proteomic features (Figure 1B) and differential phosphosignaling activity (Figure 1C).

**Figure 2:**
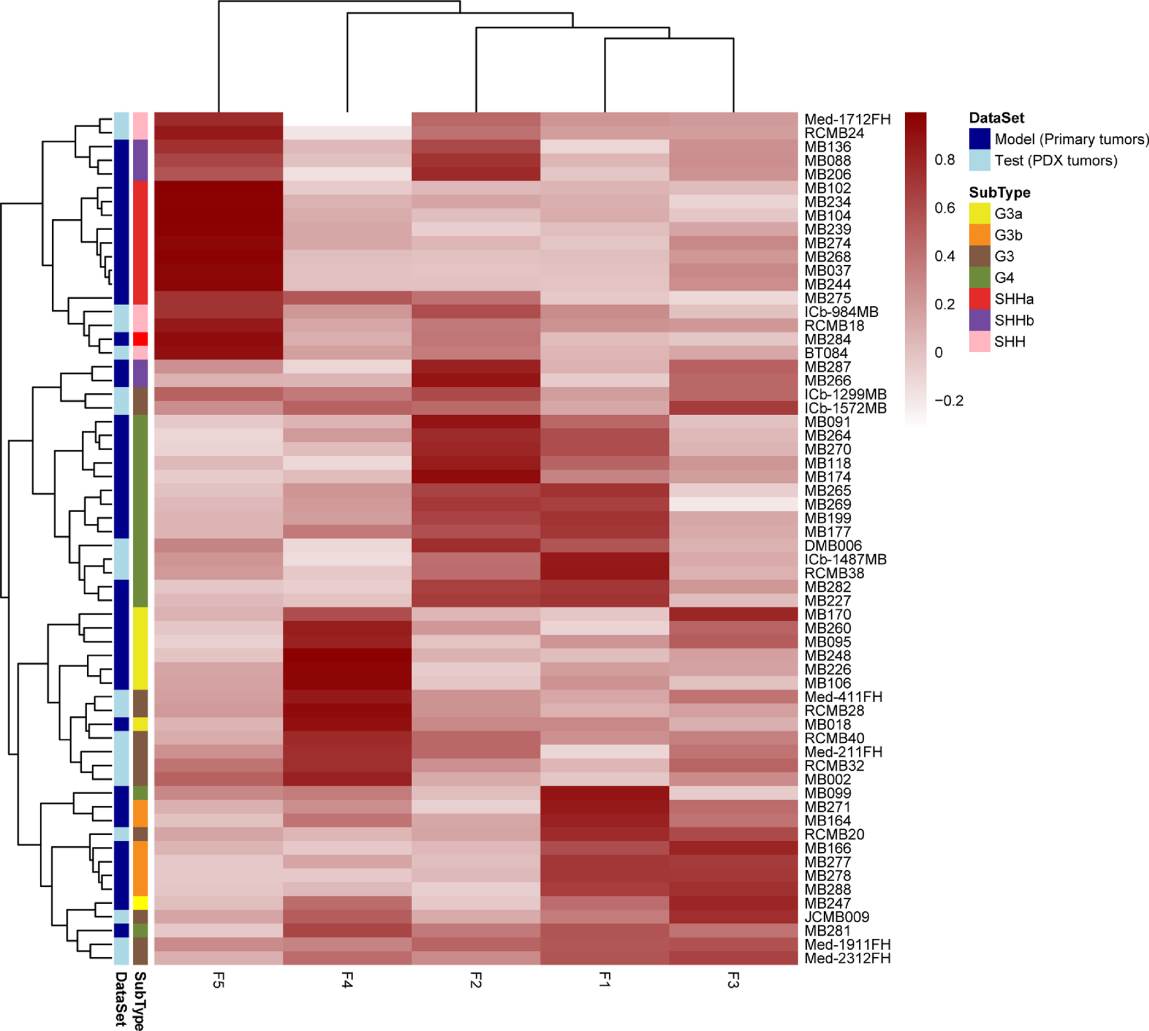
Metagene projection of PDX proteomic signatures onto the Archer proteomics dataset. Unsupervised clustering of metagene expression levels in PDX and primary human MB tumors. PDX proteomics data (test dataset) were projected onto the Archer proteomics dataset (model dataset) using the Metagene Projection module in GenePattern.

Next, we wanted to examine how the proteomic features of our MB PDXs compare to primary human MB tumors. We used metagene projection^37^ to project our MB PDX proteomics data on to the Archer et al.^8^ proteomics dataset, which includes SHHa, SHHb, G3a, G3b, and G4 primary human MB tumors. The number of metagenes was set to 5 as non-negative matrix factorization (NMF) on the Archer dataset confirmed that *k* = 5 provided optimal clustering (Figure S5). Metagene projection of our proteomics data onto the Archer dataset confirmed that the proteomic features of primary MB tumors, including proteomic signatures that distinguish different MB subgroups, are maintained in MB PDX models (Figure 2 and Table S3). Of the SHH PDXs, RCMB18, Med-1712FH, RCMB24, and BT084 most closely resembled primary SHHa tumors while ICb-984MB presented features of both SHHa and SHHb MB. RCMB32 strongly resembled G3a MB, confirming divergence of this PDX line. Of the G3 PDXs, MB002, Med-211FH, Med-411FH, RCMB28, and RCMB40 most closely resembled primary G3a MB tumors while JCMB009, Med-1911FH, Med-2312FH, ICb-1299MB, ICb-1572MB, and RCMB20 more closely resembled primary G3b tumors. Differential analysis of G3a and G3b PDXs did not identify specific pathways unique to G3b MB but rather showed that G3b PDXs displayed features also found in SHH and G4 PDXs (Figure S6). This finding is consistent with our metagene projection results that showed that G3b MB is enriched for proteomic signatures that are also found in G4 MB. Overall, our results demonstrate that the proteomic features of primary MB tumors are conserved in MB PDX models.

### Subgroup Enriched Processes and Pathways

**Figure 3:**
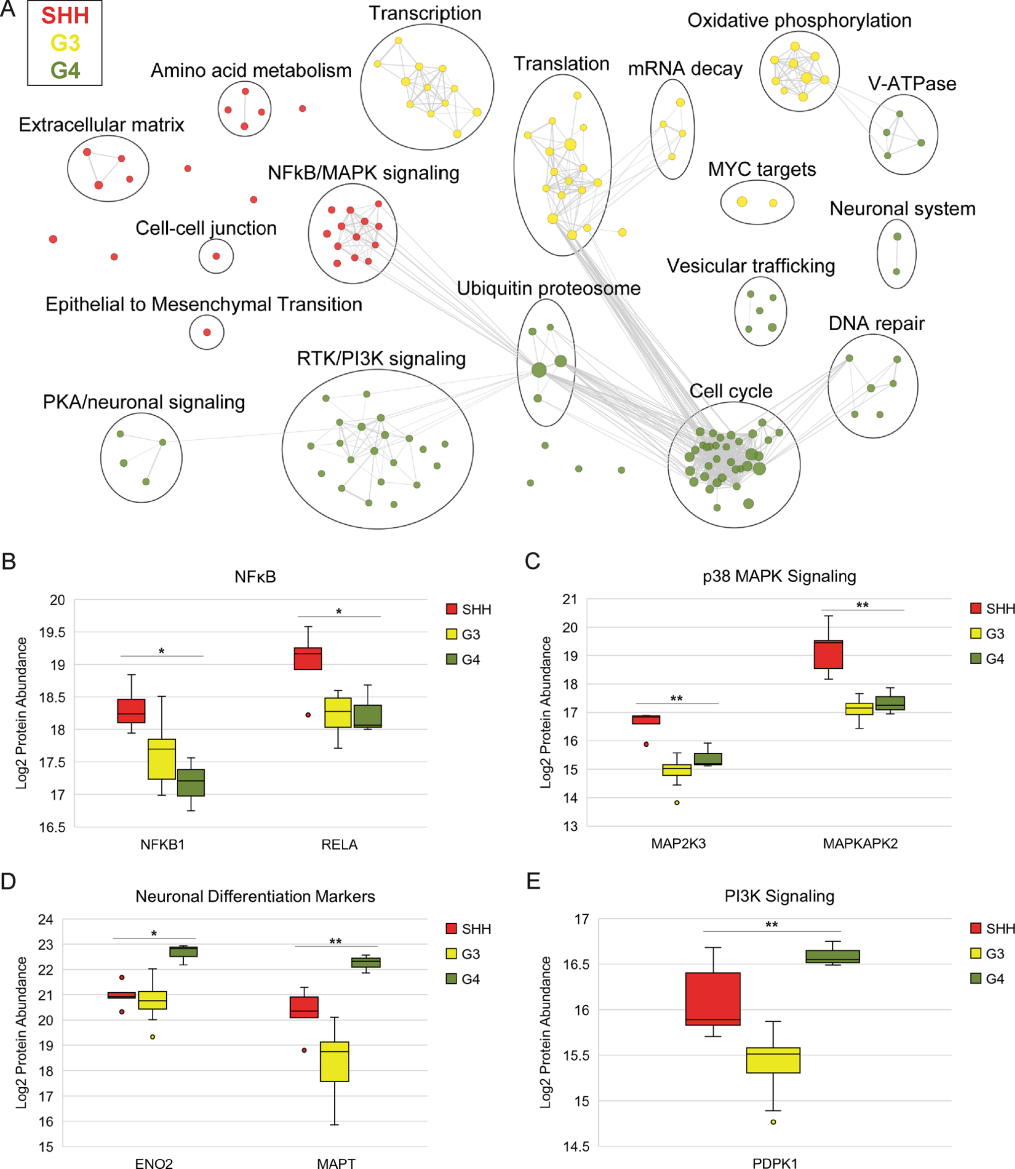
Subgroup-enriched processes and pathways. (A) Hallmark 50 and C2 canonical pathways gene sets with significant (*q* < 0.05) and positive enrichment in a given subgroup for all Gene Set Enrichment Analysis comparisons against the other two subgroups. Significant gene sets are visualized with the EnrichmentMap app in Cytoscape. Each node represents an enriched gene set, and lines connect gene sets with shared members. Nodes are colored by enriched subgroup. Node size corresponds to gene set size. (B–E) Protein abundance of NFKB1 and RELA (B), MAP2K3 and MAPKAPK2 (C), ENO2 and MAPT (D), and PDPK1 (E) in MB subgroups. *ANOVA *q* < 0.05, **ANOVA *q* < 0.01.

To identify pathways and cellular processes specifically enriched in each subgroup, we performed Gene Set Enrichment Analysis (GSEA) (Figure 3A and Table S4). SHH PDXs were enriched for pro-inflammatory signaling pathways including the NFκB and p38 MAPK signaling pathways. NFκB subunits NFKB1/p105 and RELA/p65 were significantly more abundant in SHH PDXs (Figure 3B). The p38 MAPK kinase MAP2K3/MKK3 and the p38 MAPK effector MAPKAPK2/MK2 were also significantly upregulated in SHH PDXs (Figure 3C). p38 MAPK has been shown to regulate the transcriptional activity of NFκB.^38^ Notably, SHH PDXs were also enriched for the extracellular matrix (ECM) and ECM-interacting proteins, suggesting that the tumor microenvironment plays an important role in SHH MB.

G3 PDXs were enriched for MYC target genes as well as proteins involved in transcription, RNA processing, and translation. Mitochondrial energy metabolism including the TCA cycle and oxidative phosphorylation were also upregulated in G3 PDXs.

G4 PDXs were enriched for features of differentiated neurons including vesicular transport and membrane trafficking and expressed the highest levels of neuronal differentiation markers (Figure 3D). PI3K and receptor tyrosine kinase (RTK) signaling were also enriched in G4 PDXs, which contained significantly higher levels of PDPK1 (Figure 3E). G4 PDXs were also enriched for cell cycle and DNA repair proteins. The ubiquitin proteasome system was also upregulated in G4, consistent with previous observations that proteostasis is altered in G4 MB.^7^

### Subgroup Specific Kinase Signatures

**Figure 4:**
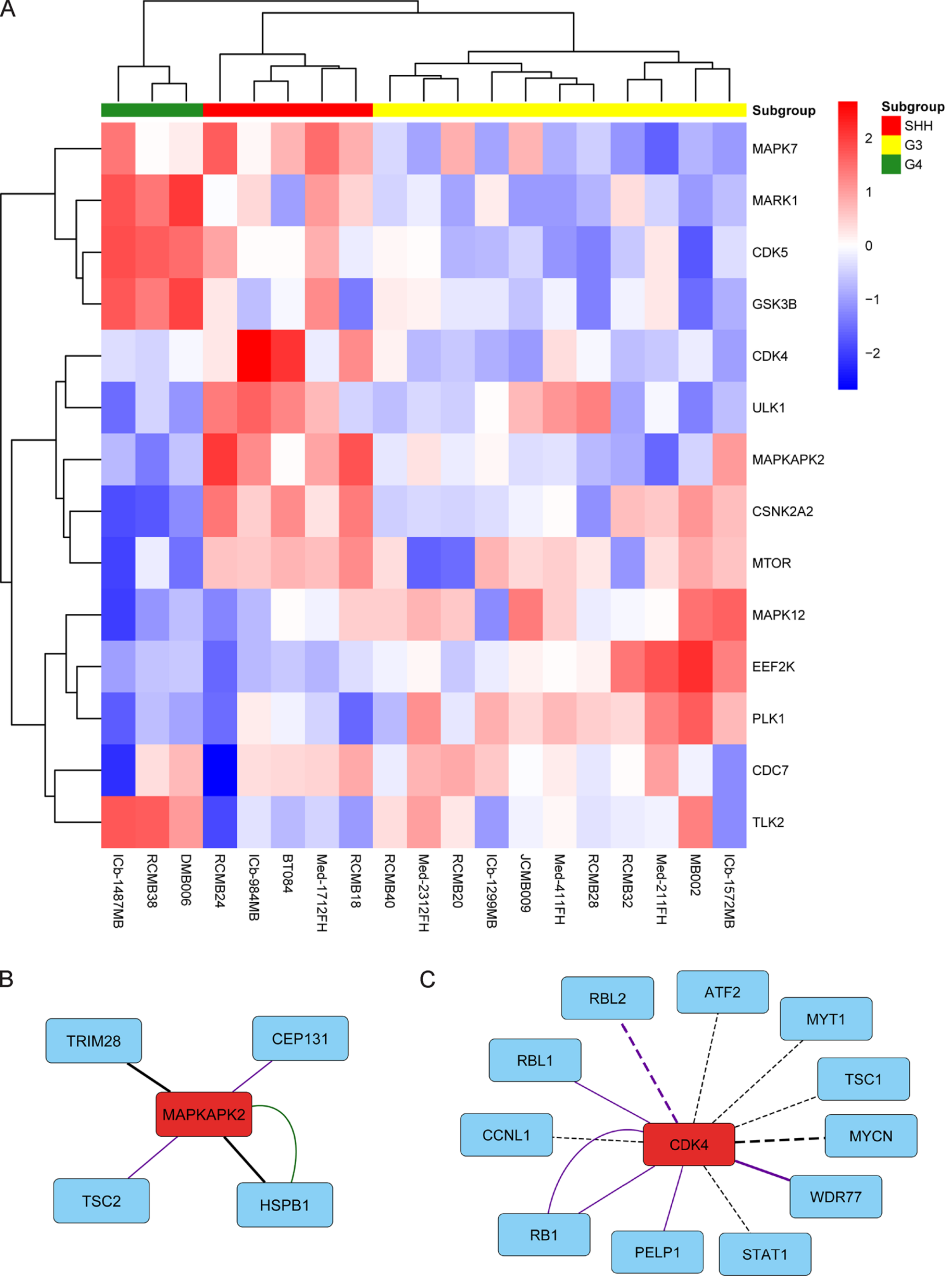
Subgroup-specific kinase activity. (A) Kinase activity of kinases with significant differential activity between subgroups (ANOVA *q* < 0.05). (B,C) Known (solid line) and predicted (dashed line) substrates of MAPKAPK2 (B) and CDK4 (C) in our dataset. Line color indicates the functional consequence of phosphorylation: green – activating; purple – regulatory; black – unknown. Phosphosites that are significantly differentially abundant between subgroups (ANOVA *q* < 0.05) are indicated with a thicker line.

To quantify kinase activity in each MB PDX, we used the human PhosphoSitePlus database^29^ and the kinase-substrate prediction tool NetworKIN^30^ to match experimentally observed and high confidence (Figure S7) predicted kinases to phosphosites in our dataset. The activity of 47 identified kinases was then scored using the machine learning algorithm IKAP^31^ (Table S5). Fourteen kinases had significant differential kinase activity between subgroups (Figure 4A).

Consistent with our GSEA analysis, SHH PDXs had significantly higher activity of the p38 MAPK effector MAPKAPK2 (Figure 4B). Notable substrates of MAPKAPK2 observed in our dataset include the heat shock protein HSPB1/HSP27 and the mTOR Complex 1 (mTORC1) regulator TSC2. MAPKAPK2 phosphorylation of HSPB1 has been shown to promote cancer cell survival in response to genotoxic stress.^39^ TSC2 is a negative regulator of mTORC1. Phosphorylation of TSC2 by MAPKAPK2 promotes 14-3-3 protein binding and negatively modulates TSC2 function.^40,41^ mTOR kinase activity was significantly upregulated in SHH PDXs, consistent with downregulation of TSC2.

CDK4 activity was also significantly elevated in SHH PDXs (Figure 4C). CDK4 is an important driver in many cancer types including medulloblastoma.^42^ In addition to observing well-known substrates of CDK4 such as RB1, we also identified high-confidence predicted substrates of CDK4. MYCN, which was amplified in three SHH PDXs, was predicted to be phosphorylated by CDK4. Other predicted substrates of CDK4 included Cyclin-L1 (CCNL1) and Myelin transcription factor 1 (MYT1).

G3 PDXs had significantly higher activity of Polo-like kinase 1 (PLK1). Inhibition of PLK1 has been shown to slow the progression of MYC-amplified medulloblastoma by promoting MYC protein degradation.^43^ GSK3B and CDK5 were specifically upregulated in G4 PDXs.

### Actinomycin D Sensitivity

**Figure 5:**
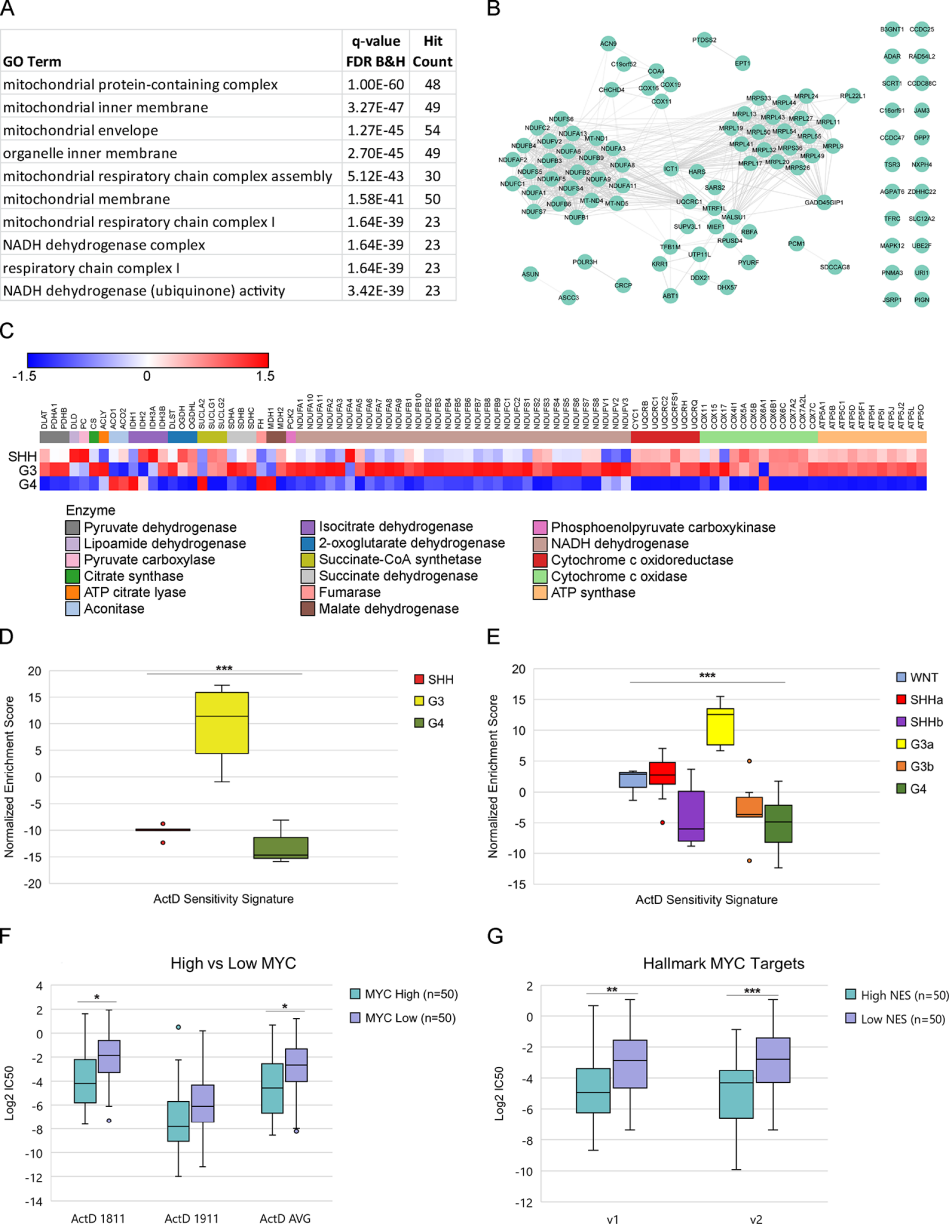
Proteomic signatures associated with actinomycin D sensitivity. (A) Protein abundances from global proteomics data were correlated with actinomycin D (ActD) sensitivity across MB PDX lines. The top 100 proteins with the strongest positive correlation to ActD sensitivity (“ActD Sensitivity Signature”) were analyzed by gene ontology (GO). The top 10 most significant GO terms are shown. (B) String network of the ActD Sensitivity Signature. (C) Relative median abundance of the TCA cycle and electron transport chain proteins in MB PDXs by subgroup. (D) Normalized enrichment scores for ActD Sensitivity Signature in MB PDXs. ***ANOVA *p* < 0.001. (E) Normalized enrichment scores for ActD Sensitivity Signature in primary human MB tumors from the Archer dataset. ***ANOVA *p* < 0.001. (F) ActD IC50 values from two independent screens (1811 and 1911) for the top 50 and bottom 50 CCLE cell lines with the highest and lowest, respectively, MYC protein abundance. *Student’s *t*-test *p* < 0.05. (G) Average ActD IC50 values for the top 50 and bottom 50 CCLE cell lines with the highest and lowest, respectively, normalized enrichment scores (NES) for the Hallmark MYC Targets v1 and Hallmark MYC Targets v2 gene sets. **Student’s *t*-test *p* < 0.01, ***Student’s *t*-test *p* < 0.001.

We previously identified actinomycin D (ActD) as a candidate therapeutic for G3 MB.^10^ To better understand the molecular basis for drug sensitivity, we correlated basal protein and phosphosite abundance with average cell viability after ActD treatment. Many of the top 100 proteins most positively associated with sensitivity to ActD (“ActD Sensitivity Signature”) were mitochondrial proteins (Figure 5A and Table S6) including members of the NADH dehydrogenase complex I of the electron transport chain (ETC). Mitochondrial ribosome proteins that synthesize core components of the ETC were also strongly correlated with ActD sensitivity (Figure 5B). ETC proteins were more highly expressed in G3PDXs compared to SHH and G4 PDXs (Figure 5C), suggesting that mitochondrial energy metabolism is upregulated in G3 MB. Notably, many of the mitochondrial proteins in the ActD Sensitivity Signature are known downstream targets of MYC.^44^ Our identified ActD Sensitivity Signature was significantly enriched in G3 PDXs as well as in G3a primary tumors from the Archer dataset^8^ (Figure 5D,E). Thus, we hypothesized that MYC-driven cancers would be more sensitive to ActD. Using previously published quantitative proteomics data^34^ and ActD IC50 values^35,36^ on Cancer Cell Line Encyclopedia (CCLE) cell lines, we found that cell lines with high MYC protein abundance were significantly (Student’s *t*-test *p* < 0.05) more sensitive to ActD than cell lines with low MYC protein abundance (Figure 5F). Additionally, CCLE cell lines with high protein abundance of known MYC target genes were also significantly more sensitive to ActD than cell lines with low MYC target protein abundance (Figure 5G). These results suggest that ActD may also be efficacious against other MYC-driven cancers.

## Discussion

Through proteomic and phosphoproteomic characterization of orthotopic PDX models of MB, we show that SHH, G3, and G4 MB PDXs display distinct proteomic features and exemplify many characteristics of primary human MB tumors. A major advantage of orthotopic PDX models is that they allow for functional experiments to study MB tumor biology. Multiple studies have shown that orthotopic PDX models of pediatric brain tumors maintain the histological and genomic features of the primary tumors they are derived from.^46,47^ However, serial passaging of xenograft tumors may inadvertently cause mouse-specific tumor evolution, especially in a heterologous environment.^48^ The observed divergence of the SHH PDX RCMB32 and clustering with G3 PDXs may be due to the expansion of select subclones from the original tumor population. Our metagene projection analysis indicated that the proteomic signatures identified in these MB PDXs are closely aligned with those of primary human MB tumors, emphasizing the utility of these models.

Our proteomic analysis showed that the G3 PDXs analyzed in this study primarily resemble G3a MB. G3a MB is characterized by activation of MYC,^8^ and eight G3 PDXs have amplification of MYC. MYC regulated processes including transcription, translation, and mitochondrial function were significantly upregulated in G3 PDXs. While some G3 PDXs did show enrichment for a G3b signature identified in primary human MB tumors, we were unable to resolve the key pathways driving G3b MB. This is likely due to the overrepresentation of MYC-amplified G3 PDXs in our cohort. Notably, G3 PDXs with high G3b signature expression also expressed higher levels of a signature also found in G4 MB tumors. Single cell sequencing studies have suggested that G3 and G4 MBs differ in differentiation state, with G3 MB existing primarily as undifferentiated cells and G4 MBs as mostly differentiated neuronal cells.^49^ Indeed, our G4 PDXs displayed proteomic features typical of differentiated neurons including upregulated vesicular trafficking and endocytosis as well as high abundance of neuronal differentiation markers. Another phosphoproteomic study reported two distinct signaling states of MB, with a subset of G3 tumors displaying a MYC signature and another subset of G3 tumors displaying a neuronal signature similar to G4 MB.^50^ G3b MB may represent an intermediate state between undifferentiated MYC-driven G3a and the more differentiated neuronal phenotype of G4.

SHH PDXs were enriched for several signatures that suggest enhanced interaction with the tumor microenvironment. Compared to other MB subgroups, SHH MB tumors have increased stromal cell activity.^51^ Stromal astrocytes have been shown to promote SHH MB tumorigenesis by secreting the sonic hedgehog ligand as well as extracellular matrix,^52,53^ which was more abundant in SHH PDXs. Pro-inflammatory signaling via NFκB and p38 MAPK was also upregulated in SHH PDXs. NFκB activation has been observed in stem-like cells in SHH MB,^54^ and up-regulation of genes in the NFκB pathway has been linked to poor prognosis in G4 MB.^55^

We identified a strong correlation between high abundance of mitochondrial proteins and ActD sensitivity. MYC is a key regulator of mitochondrial biogenesis.^56,57^ We found that CCLE cell lines with high abundance of MYC and MYC target genes were more sensitive to ActD. Mechanistically, ActD intercalates into GC-rich regions of DNA and blocks transcriptional elongation by RNA polymerases.^58^ ActD has been shown to bind to regions of the MYC promoter and repress MYC expression.^59,60^ MYC target genes as well as transcription and RNA processing proteins were highly upregulated in G3 MB PDXs. Additionally, ActD preferentially inhibits RNA Pol I transcription, which synthesizes key ribosomal RNAs.^61,62^ Mitochondrial transcription and translation are required for the synthesis of large hydrophobic proteins in the ETC.^63^ In our prior drug screen,^10^ the mitochondrial complex I inhibitor rotenone was efficacious against most G3 PDXs. Disruption of mitochondrial oxidative phosphorylation by inhibitors such as rotenone has been shown to downregulate MYC expression.^64^ Our results suggest that the efficacy of ActD in G3 MB stems from its ability to inhibit global transcription and, possibly, mitochondrial function. Mitochondrial function may be a potential therapeutic vulnerability in G3 MB.

## Conclusions

Here, we have provided a comprehensive proteomic and phosphoproteomic characterization of commonly studied PDX models of SHH, G3, and G4 MB. Our study demonstrates that orthotopic PDX MB models recapitulate many features of primary MB tumors and identifies upregulated pathways and kinases in each proteomic subgroup that may serve as candidate targets for the development of new therapies. Additionally, we show that proteomics data can be used to identify signatures associated with drug sensitivity and find a significant association between MYC and Actinomycin D sensitivity. Given the heterogeneous nature of MB, understanding how proteomic signatures correlate with drug sensitivity will allow for more targeted approaches to MB treatment.

## Supporting Information Available

Mouse cell infiltration in medulloblastoma PDXs (Figure S1); unsupervised hierarchical clustering of global deep expression proteomics and phosphoproteomics data (Figure S2); principal component analysis of global deep expression proteomics and phosphoproteomics data (Figure S3); missing data in global deep expression proteomics and phosphoproteomics datasets (Figure S4); non-negative matrix factorization on the Archer proteomics dataset (Figure S5); differentially abundant proteins in G3a vs G3b PDXs (Figure S6); NetworKIN confidence scores (Figure S7) (PDF)
PDX lines (Table S1) (XLSX)
TMT channel assignments (Table S2) (XLSX)
Metagene Projection cluster assignments (Table S3) (XLSX)
Gene Set Enrichment Analysis (GSEA) results (Table S4) (XLSX)
All kinase activity (Table S5) (XLSX)
Actinomycin D sensitivity signature gene ontology (GO) results (Table S6) (XLSX)

## ABBREVIATIONS

MB: medulloblastoma
WNT: wingless
SHH: sonic hedgehog
G3: Group 3
G4: Group 4
PDX: patient-derived xenograft
ActD: actinomycin D
PSM: peptide spectrum match
NSG: NOD-SCID IL2R-gamma null
TMT: tandem mass tag
FDR: false discovery rate
SL: sample loading
IRS: internal reference standard
TMM: trimmed mean of *m* samples
*C*: Cochran C test statistic
GSEA: Gene Set Enrichment Analysis
MSigDB: Molecular Signatures Database
PSP: PhosphoSitePlus
IKAP: Inference of Kinase Activities from Phosphoproteomics data
MCC: Matthew’s correlation coefficient
GO: gene ontology
NMF: non-negative matrix factorization
mTORC1: mTOR Complex 1
RTK: receptor tyrosine kinase
ETC: electron transport chain
TCA: tricarboxylic acid
