# Supplementary material for: Subgroup-Enriched Pathways and Kinase Signatures in Medulloblastoma Patient-Derived Xenografts
Source: J Proteome Res. 2022 Aug 17;21(9):2124–36. doi: 10.1021/acs.jproteome.2c00203 (PMC9442791; doi:10.1021/acs.jproteome.2c00203)
Supplement: Supplementary file 1 — pr2c00203_si_001.pdf [file pr2c00203_si_001.pdf]

SUPPORTING INFORMATION

Subgroup-enriched pathways and kinase signatures in medulloblastoma patient-derived xenografts

Kristin L. Leskoske[1], *Krystine Garcia-Mansfield*[1,2], *Ritin Sharma*[1,2], *Aparna Krishnan*[1], *Jessica M. Rusert*[3], *Jill P. Mesirov*[4,5], *Robert J. Wechsler-Reya*[3], *Patrick Pirrotte*[1,2]

[1]Cancer and Cell Biology Division, Translational Genomics Research Institute, Phoenix, AZ, 85004, USA

[2]Integrated Mass Spectrometry Shared Resource, City of Hope Comprehensive Cancer Center, Duarte, CA, 91010, USA

[3]Tumor Initiation and Maintenance Program, NCI-Designated Cancer Center, Sanford Burnham Prebys Medical Discovery Institute, La Jolla, CA, 92037, USA

[4]Department of Medicine, University of California San Diego, La Jolla, CA, 92093, USA

[5]Moores Cancer Center, University of California San Diego, La Jolla, CA, 92093, USA

Table of Contents

Figure S1: Mouse cell infiltration in medulloblastoma PDXs.

Figure S2: Unsupervised clustering of global deep expression proteomics and phosphoproteomics data.

Figure S3: Principal component analysis of global deep expression proteomics and phosphoproteomics data.

Figure S4: Missing data in global deep expression proteomics and phosphoproteomics data.

Figure S5: Non-negative matrix factorization on Archer proteomics data.

Figure S6: Differentially abundant proteins in G3a vs G3b medulloblastoma PDXs.

Figure S7: NetworKIN confidence scores for kinase-substrate predictions.

Table S1 (.xlsx): PDX lines.

Table S2 (.xlsx): TMT channel and batch assignments.

Table S3 (.xlsx): Metagene Projection cluster assignments.

Table S4 (.xlsx): Gene Set Enrichment Analysis (GSEA) results.

Table S5 (.xlsx): All scored kinase activity.

Table S6 (.xlsx): Actinomycin D sensitivity signature Gene Ontology results.

Figure S1

A

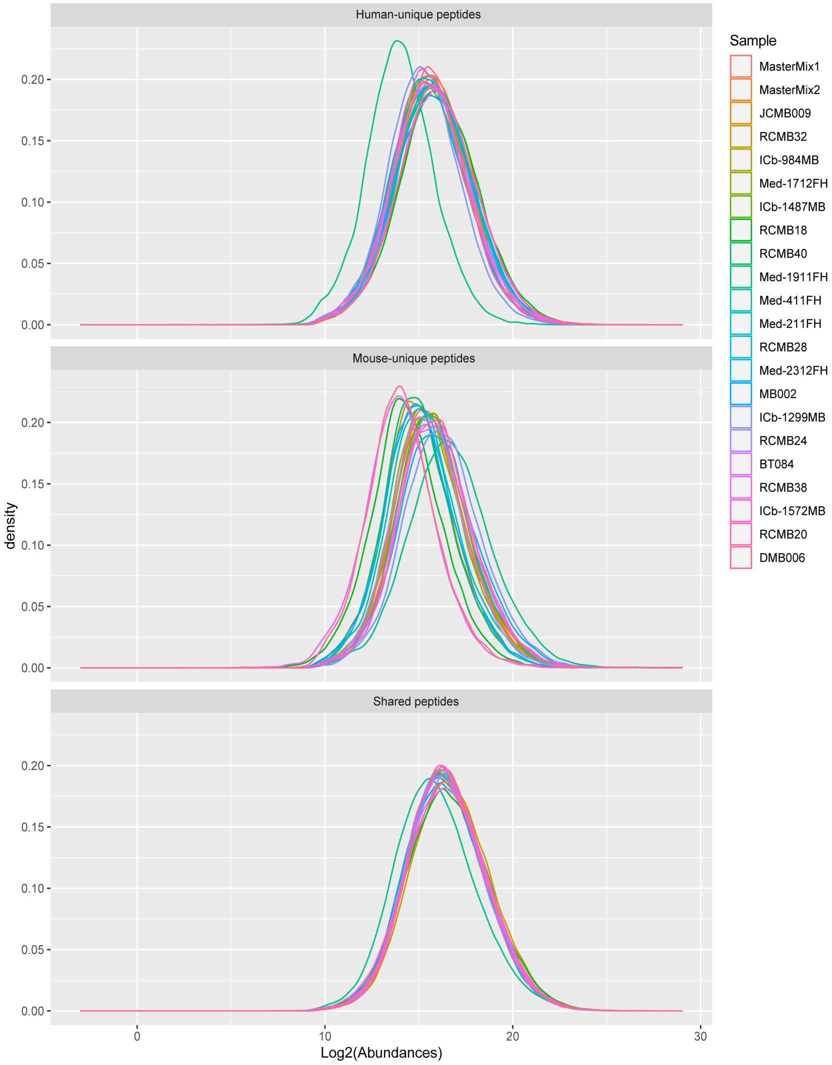

Human-unique peptides
Mouse-unique peptides
Shared peptides
density
Log2(Abundances)
0.00
0.05
0.10
0.15
0.20
0
10
20
30
Sample
MasterMix1
MasterMix2
JCMB009
RCMB32
ICb-984MB
Med-1712FH
ICb-1487MB
RCMB18
RCMB40
Med-1911FH
Med-411FH
Med-211FH
RCMB28
Med-2312FH
MB002
ICb-1299MB
RCMB24
BT084
RCMB38
ICb-1572MB
RCMB20
DMB006

B

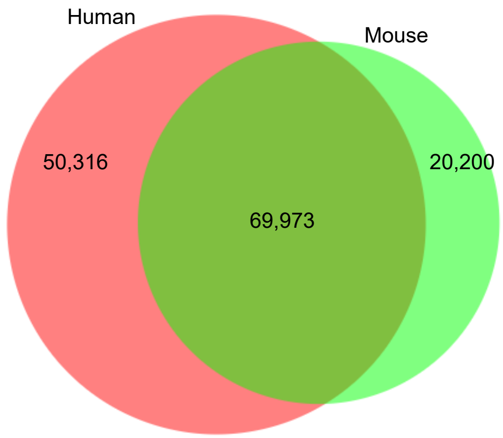

Human
Mouse
50,316
69,973
20,200

Figure S1: Mouse cell infiltration in medulloblastoma PDXs. (A) Abundance distribution of human-unique, mouse-unique, and shared (human or mouse) peptides in global deep expression proteomics dataset. (B) Overlap of human and mouse peptides from *(A)*.

Figure S2

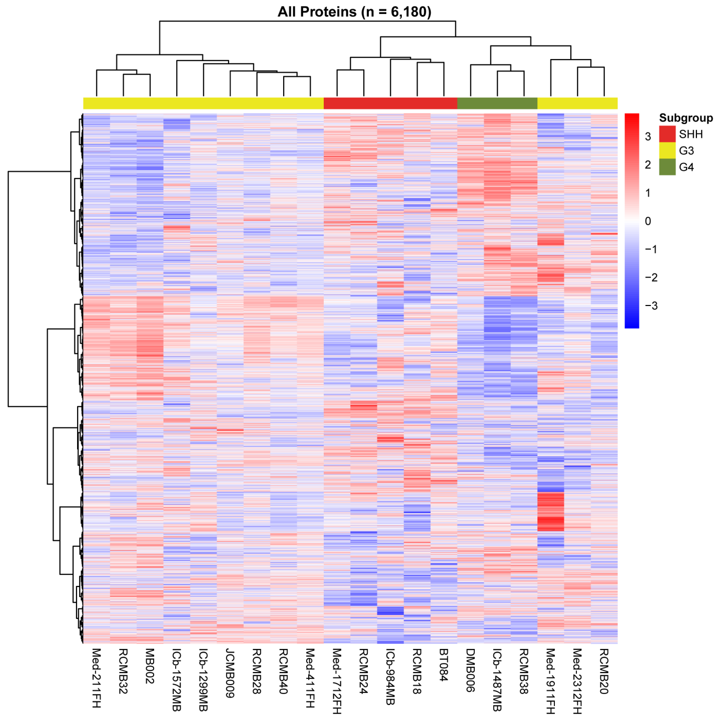
All Proteins (n = 6,180)
Subgroup
SHH
G3
G4
3
2
1
0
−1
−2
−3
Med-211FH
RCMB32
MB002
ICb-1572MB
ICb-1299MB
JCMB009
RCMB28
RCMB40
Med-411FH
Med-1712FH
RCMB24
ICb-984MB
RCMB18
BT084
DMB006
ICb-1487MB
RCMB38
Med-1911FH
Med-2312FH
RCMB20

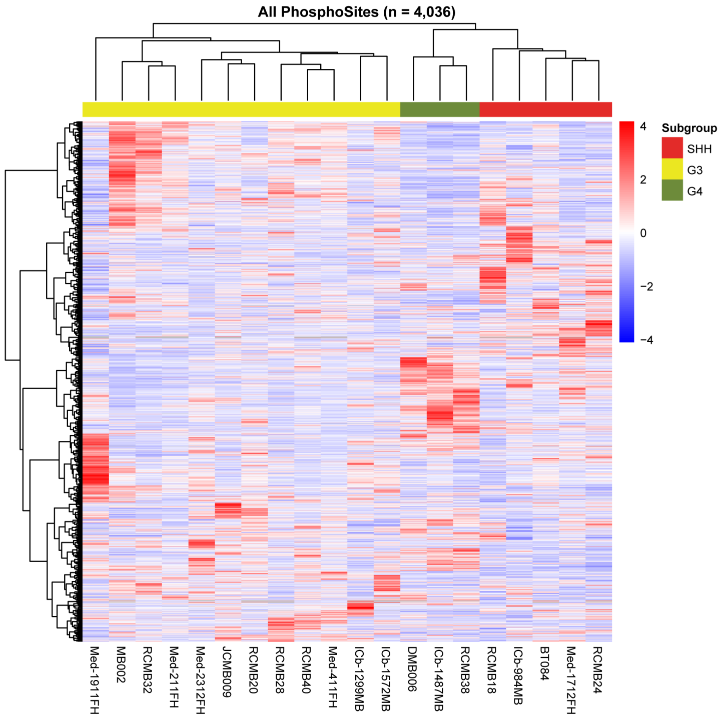
All PhosphoSites (n = 4,036)
Subgroup
SHH
G3
G4
4
2
0
−2
−4
Med-1911FH
MB002
RCMB32
Med-211FH
Med-2312FH
JCMB009
RCMB20
RCMB28
RCMB40
Med-411FH
ICb-1299MB
ICb-1572MB
DMB006
ICb-1487MB
RCMB38
RCMB18
ICb-984MB
BT084
Med-1712FH
RCMB24

Figure S2: **Unsupervised hierarchical clustering** (Euclidean, Ward) of global deep expression proteomics (*left*) and phosphoproteomics (*right*) data.

Figure S3

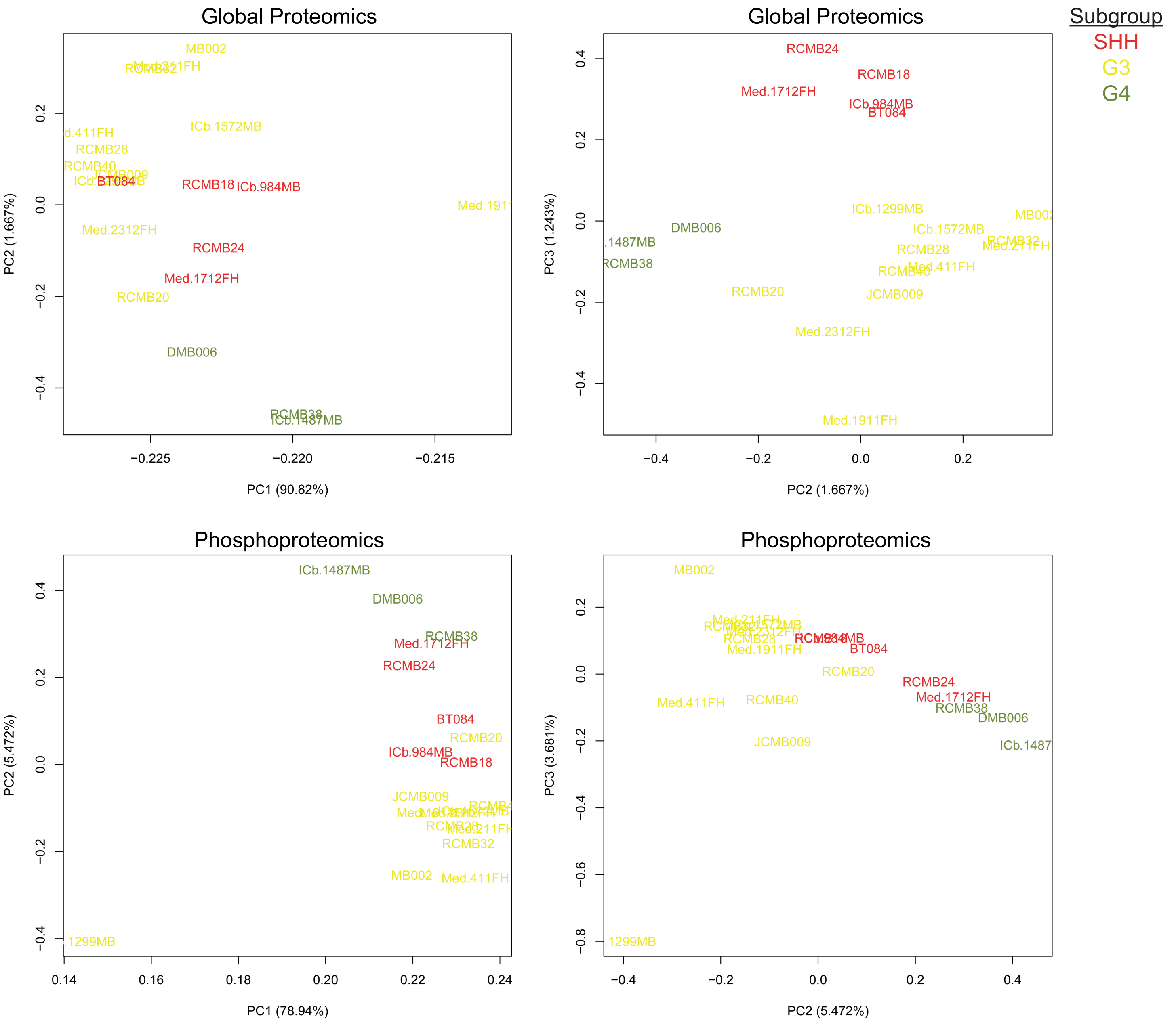

Global Proteomics
PC1 (90.82%)
PC2 (1.667%)
Global Proteomics
PC2 (1.667%)
PC3 (1.243%)
Subgroup
SHH
G3
G4
Phosphoproteomics
PC1 (78.94%)
PC2 (5.472%)
Phosphoproteomics
PC2 (5.472%)
PC3 (3.681%)

Figure S3: Principal component analysis of global deep expression proteomics (*top*) and phosphoproteomics (*bottom*) data.

Figure S4

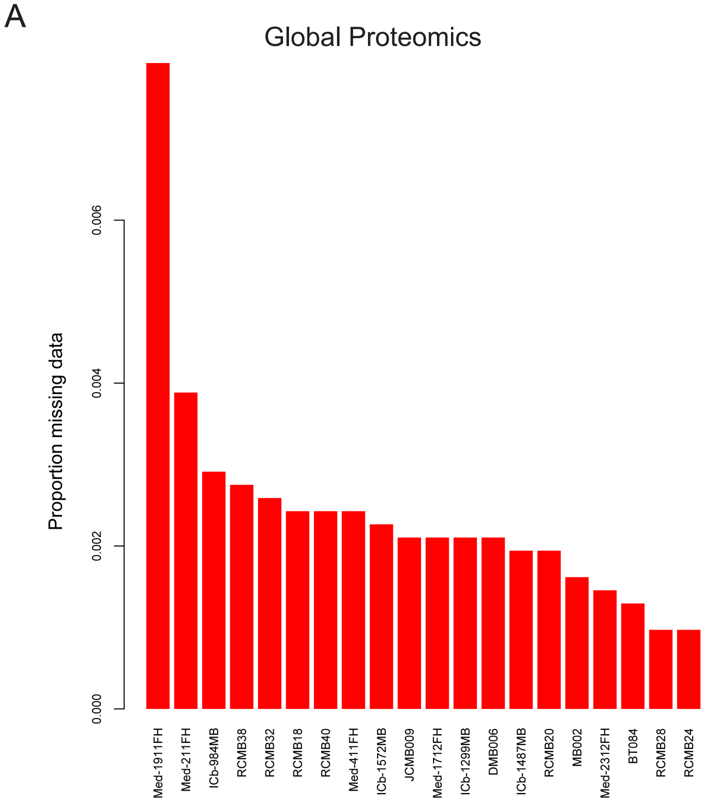
A
Global Proteomics
Proportion missing data
0.000
0.002
0.004
0.006
Med-1911FH
Med-211FH
ICb-984MB
RCMB38
RCMB32
RCMB18
RCMB40
Med-411FH
ICb-1572MB
JCMB009
Med-1712FH
ICb-1299MB
DMB006
ICb-1487MB
RCMB20
MB002
Med-2312FH
BT084
RCMB28
RCMB24

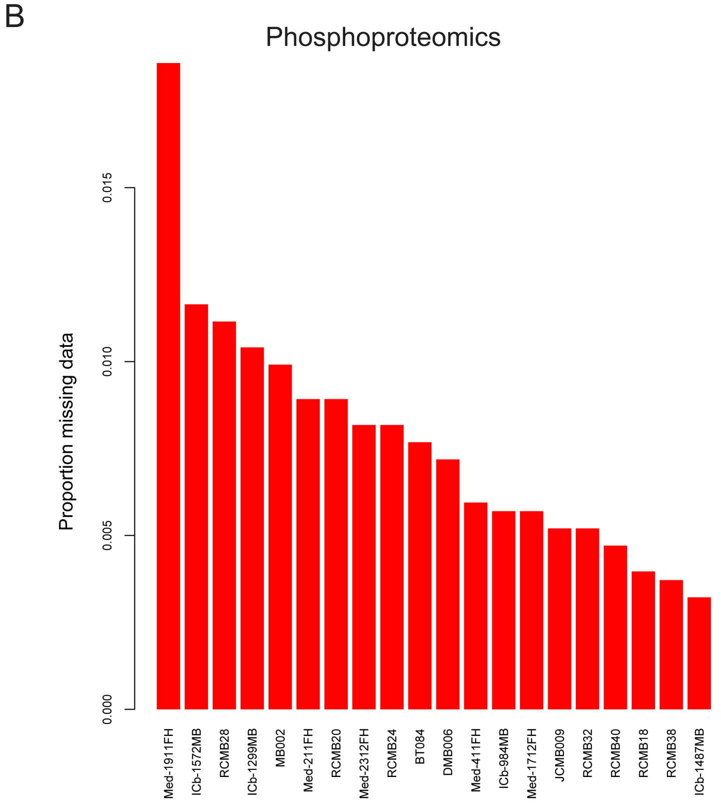
B
Phosphoproteomics
Proportion missing data
0.000
0.005
0.010
0.015
Med-1911FH
ICb-1572MB
RCMB28
ICb-1299MB
MB002
Med-211FH
RCMB20
Med-2312FH
RCMB24
BT084
DMB006
Med-411FH
ICb-984MB
Med-1712FH
JCMB009
RCMB32
RCMB40
RCMB18
RCMB38
ICb-1487MB

Figure S4: Proportion of missing data in global deep expression proteomics (*A*) and phosphoproteomics (*B*) data.

Figure S5

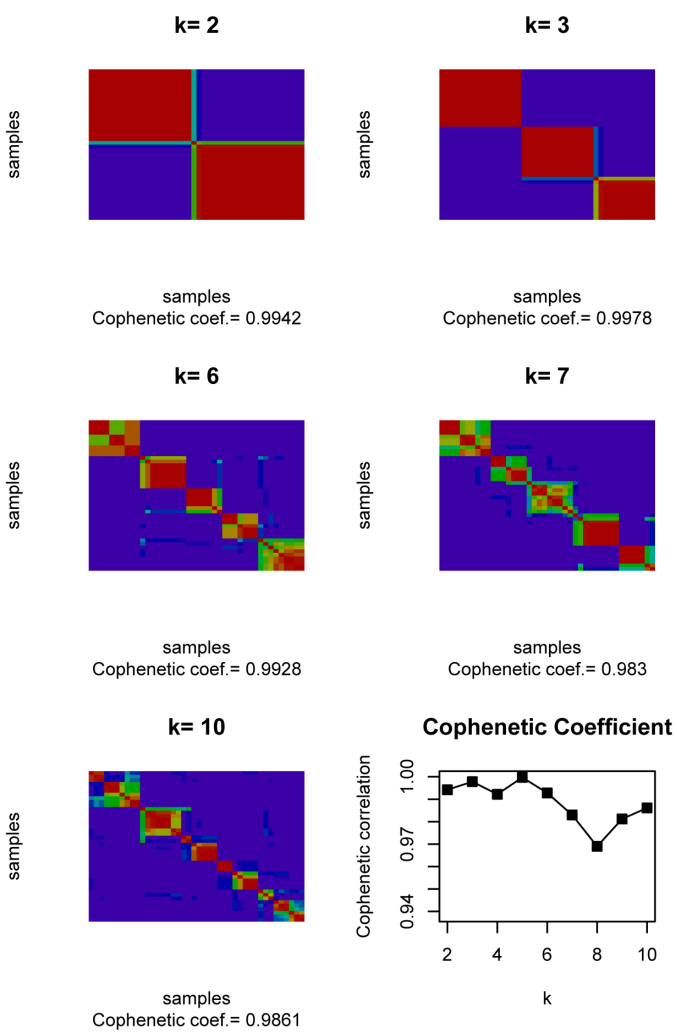
k= 2
samples
samples
Cophenetic coef.= 0.9942
k= 3
samples
samples
Cophenetic coef.= 0.9978
k= 4
samples
samples
Cophenetic coef.= 0.9922
k= 5
samples
samples
Cophenetic coef.= 0.9997
k= 6
samples
samples
Cophenetic coef.= 0.9928
k= 7
samples
samples
Cophenetic coef.= 0.983
k= 8
samples
samples
Cophenetic coef.= 0.969
k= 9
samples
samples
Cophenetic coef.= 0.9812
k= 10
samples
samples
Cophenetic coef.= 0.9861
Cophenetic Coefficient
Cophenetic correlation
0.94
0.97
1.00
2
4
6
8
10
k

Figure S5: Non-negative matrix factorization on Archer proteomics dataset. The Archer proteomics dataset (WNT samples removed) was inputted into the NMF module in GenePattern. k = 2 through 10 were tested and k = 5 proved optimal with the highest cophenetic coefficient.

Figure S6

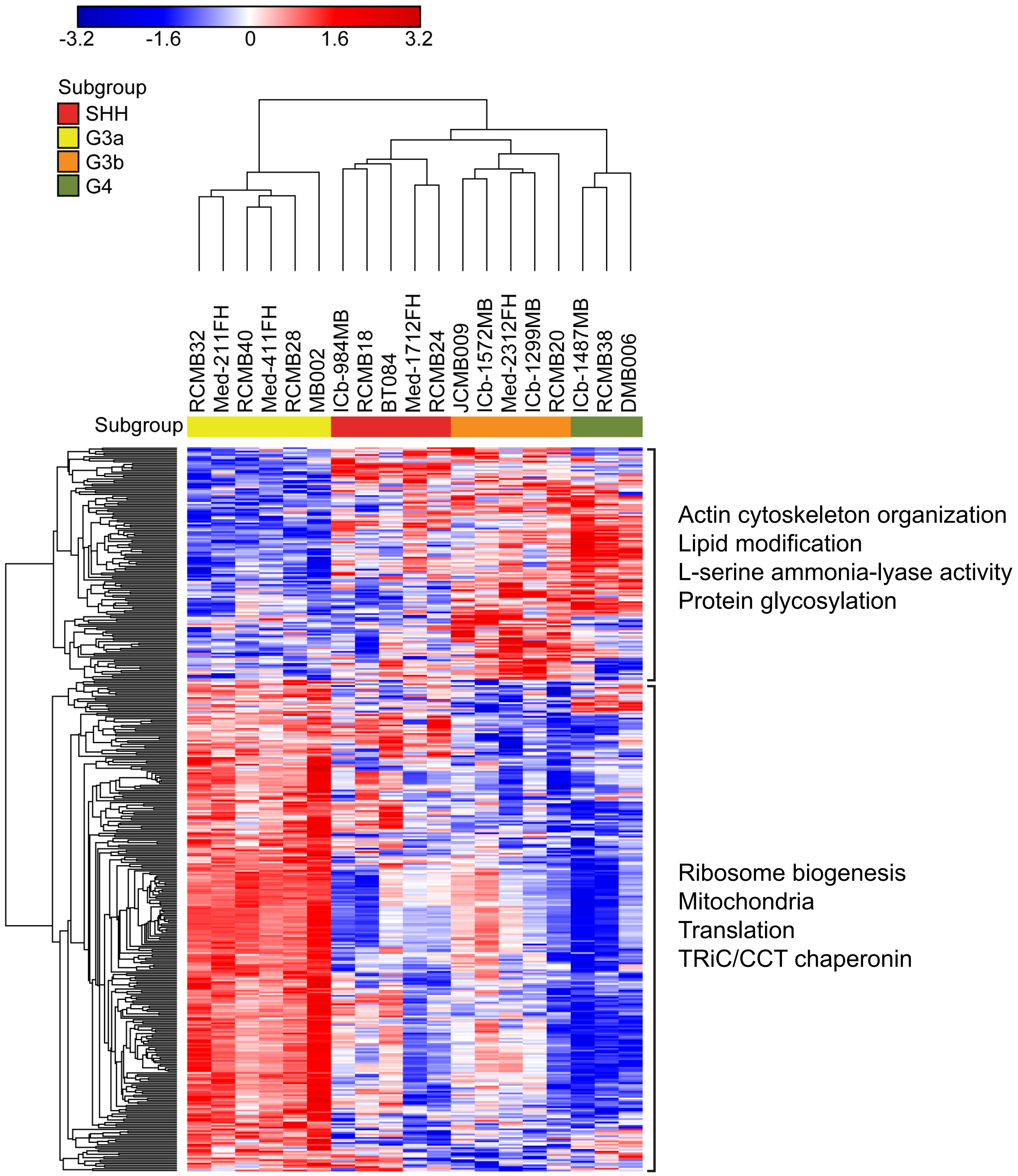
-3.2
-1.6
0
1.6
3.2
Subgroup
SHH
G3a
G3b
G4
RCMB32
Med-211FH
RCMB40
Med-411FH
RCMB28
MB002
ICb-984MB
RCMB18
BT084
Med-1712FH
RCMB24
JCMB009
ICb-1572MB
Med-2312FH
ICb-1299MB
RCMB20
ICb-1487MB
RCMB38
DMB006
Subgroup
Actin cytoskeleton organization
Lipid modification
L-serine ammonia-lyase activity
Protein glycosylation
Ribosome biogenesis
Mitochondria
Translation
TRiC/CCT chaperonin

Figure S6: Differentially abundant proteins in G3a vs G3b MB PDXs. G3 PDXs were assigned to either G3a or G3b based on metagene signature expression. An ANOVA with Tukey post-hoc was used to identify significant proteins between G3a and G3b PDXs.

Figure S7

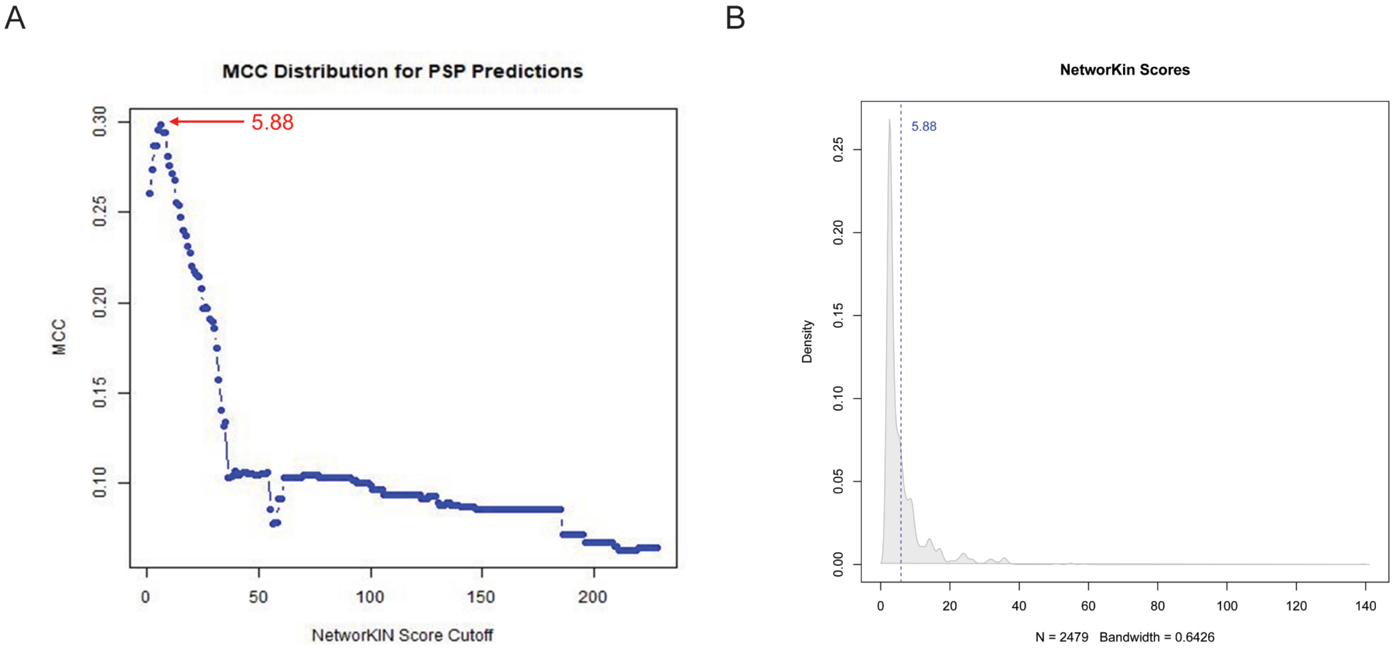
A
B
MCC Distribution for PSP Predictions
5.88
MCC
0.10
0.15
0.20
0.25
0.30
0
50
100
150
200
NetworKIN Score Cutoff
NetworKin Scores
5.88
Density
0.00
0.05
0.10
0.15
0.20
0.25
0
20
40
60
80
100
120
140
N = 2479 Bandwidth = 0.6426

Figure S7: NetworKIN confidence scores. (A) Distribution of Matthew's Correlation Coefficient (MCC) for different NetworKIN confidence score cutoffs. To determine an optimal NetworKIN score cutoff, NetworKIN was used to predict kinases for phosphosites that have known modifying kinases annotated in the human phosphosite plus database (PSP). PSP annotated kinase-substrate predictions were considered either true positives or false negatives if the NetworKIN score fell, respectively, above or below the cutoff. Likewise, kinase-substrate predictions not in the PSP database were considered either true negatives or false positives if the NetworKIN score was, respectively, below or above the cutoff. A NetworKIN score of 5.88 provided the maximum MCC. (B) Distribution of NetworKIN confidence scores for kinase-substrate predictions in the medulloblastoma phosphoproteomics dataset. Predictions with a NetworKIN confidence score greater than 5.88 were used for analysis.
